# Bone preserving level of osteotomy in short-stem total hip arthroplasty does not influence stress shielding dimensions – a comparing finite elements analysis

**DOI:** 10.1186/s12891-017-1702-2

**Published:** 2017-08-07

**Authors:** Rene Burchard, Sabrina Braas, Christian Soost, Jan Adriaan Graw, Jan Schmitt

**Affiliations:** 1 Department of Health, University of Witten/Herdecke, Witten, Germany; 2 Department of Trauma and Orthopaedic Surgery, Kreisklinikum Siegen, Siegen, Germany; 3 Department of Orthopaedics and Rheumatology, University of Marburg, Marburg, Germany; 4 Department of Statistics an Econometrics, University of Siegen, Siegen, Germany; 5 Department of Anesthesiology and Operative Intensive Care Medicine (CCM, CVK), Charité – Universitätsmedizin Berlin, Freie Universität Berlin, Humboldt-Universität zu Berlin, and Berlin Institute of Health, Campus Virchow-Klinikum, Berlin, Germany; 6 Department of Orthopaedics and Trauma Surgery, Lahn-Dill-Kliniken Wetzlar, Wetzlar, Germany

**Keywords:** Stress shielding, Short-stem, THA, Hip, Bone remodelling, Osteotomy level

## Abstract

**Background:**

The main objective of every new development in total hip arthroplasty (THA) is the longest possible survival of the implant. Periprosthetic stress shielding is a scientifically proven phenomenon which leads to inadvertent bone loss. So far, many studies have analysed whether implanting different hip stem prostheses result in significant preservation of bone stock. The aim of this preclinical study was to investigate design-depended differences of the stress shielding effect after implantation of a selection of short-stem THA-prostheses that are currently available.

**Methods:**

Based on computerised tomography (CT), a finite elements (FE) model was generated and a virtual THA was performed with different stem designs of the implant. Stems were chosen by osteotomy level at the femoral neck (collum, partial collum, trochanter sparing, trochanter harming). Analyses were performed with previously validated FE models to identify changes in the strain energy density (SED).

**Results:**

In the trochanteric region, only the collum-type stem demonstrated a biomechanical behaviour similar to the native femur. In contrast, no difference in biomechanical behaviour was found between partial collum, trochanter harming and trochanter sparing models. All of the short stem-prostheses showed lower stress-shielding than a standard stem.

**Conclusion:**

Based on the results of this study, we cannot confirm that the design of current short stem THA-implants leads to a different stress shielding effect with regard to the level of osteotomy. Somehow unexpected, we found a bone stock protection in metadiaphyseal bone by simulating a more distal approach for osteotomy. Further clinical and biomechanical research including long-term results is needed to understand the influence of short-stem THA on bone remodelling and to find the optimal stem-design for a reduction of the stress shielding effect.

**Electronic supplementary material:**

The online version of this article (doi:10.1186/s12891-017-1702-2) contains supplementary material, which is available to authorized users.

## Background

During the last two decades, total hip arthroplasty (THA) has undergone significant technical changes. On the one hand, fixation of the implant has moved from cemented to cementless techniques and on the other hand (since the Mayo® stem) the development of short-stem prostheses has been established across the board [1, 2]. The main objective of every new development in THA is the longest possible survival of implant and bone-implant-interface. Apart from polyethylene abrasion, periprosthetic stress shielding is a scientifically proven phenomenon which leads to an inadvertent bone remodelling process [3–7]. While cementless procedures were developed to simplify stem revision, short stem designs were made to imitate a physiological stem behaviour and to reduce bone remodelling processes [8]. Therefore, almost every implant company offers at least one femoral short-stem-prosthesis in their THA product line.

**Fig. 1:**
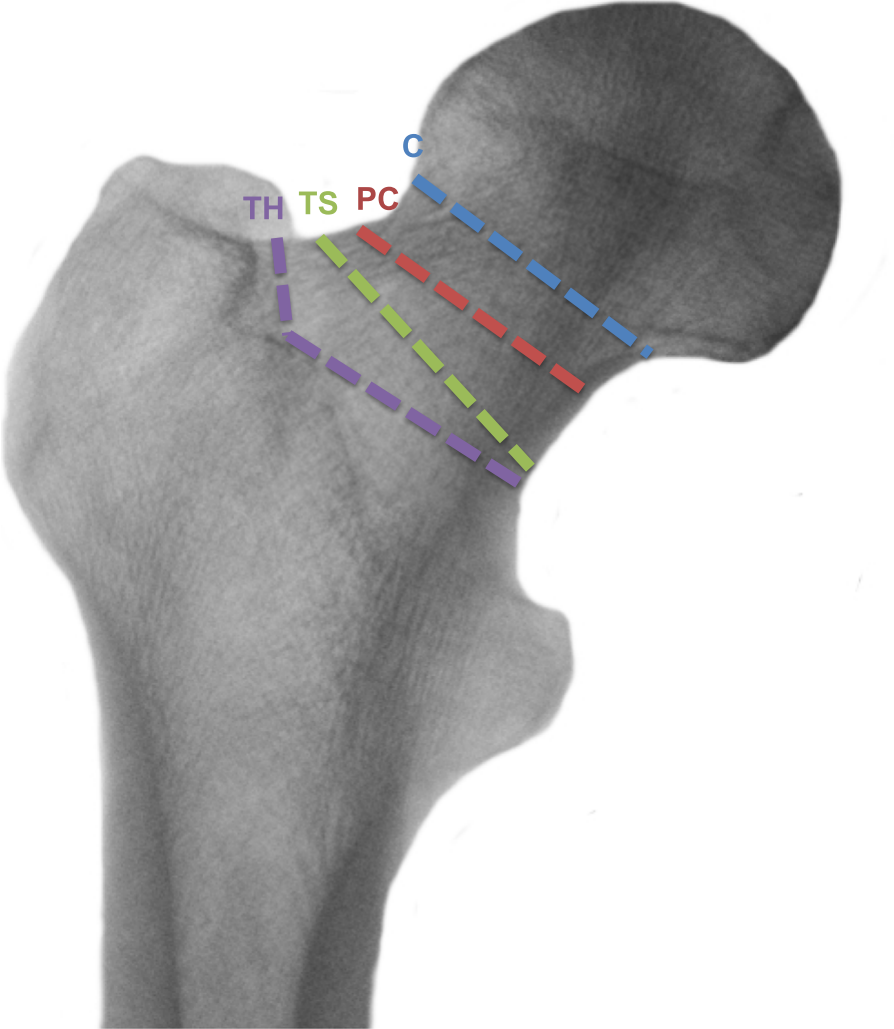
Osteotomy levels of the different stem types (collum (C), partial collum (PC), trochanter sparing (TS), and trochanter harming (TH)

Falez and colleagues have developed a classification of commercially available short hip stem prostheses referring to the level of osteotomy during THA (collum, partial collum, trochanter sparing, trochanter harming) (Fig. 1) [9]. They have extended the original classification from Feyen and Shimmin [10]. So far, many authors have analysed whether implanting the different hip stem prostheses result in significant preservation of bone stock. Preservation of bone stock is a relevant process for secure ingrowth of a standard stem in case revision surgery is needed after short stem implantation [6, 11–14].

The aim of this preclinical study was to investigate if there are short-stem-design-depended differences of the stress shielding effects in each different type of short-stem THA implant according to the classification of Falez and colleagues. Using a previously validated data set, THA-stem dependent stress-shielding effects were analysed by virtual hip stem implantation within the framework of a finite element analysis (FEA) [15].

## Methods

Based on computerised tomography (CT) by previous investigations a validated set of an in vivo scanned right femur of a female subject at the age of 75 was examined [15]. The scanner setting (Somatom® Plus-4, Siemens, Erlangen, Germany) was 140 kV, 206 mA, 17 s, spiral algorithm with recalculated slice thickness of 2 mm and a 512 × 512 pixel resolution.

The CT voxels were transferred to finite elements (FE) on a scale of 1:1 using the full information to generate the femoral model with an identical resolution of 0.66 m × 0.66 mm × 2 mm (FE and CT). The number of elements was approximately 250,000. Weinans et al. showed that differences of the stress shielding effect are independent to the density-modulus-relationship while using the same procedure for each investigation [16]. Therefore, the CT Hounsfield values (HU) were converted linearly into elastic moduli while bone was located between 170 and 1799 HU and transferred linearly to elastic moduli between 1500 and 15,000 MPa [17, 18].

Table 1 shows the different stem prostheses with their specifications including manufacturer, applied size, size-specific length, largest depth of the stem body and classification type by Falez and colleagues. The different stem designs were realised through a virtual implantation using the FE software Ansys® (Ansys 14.5.7, Ansys Inc., Canonsburg, USA). A geometrical matrix of each stem was generated and an automatic algorithm selected all elements of the bony model that belong to the matrix information [19]. The elements were assigned to an elastic modulus value of titanium alloy (110,000 MPa). FEA was performed on the cluster of the University of Siegen which provides 272 Intel-Xeon®-CPUs, 6.4 TB Working Space and 40 TB physical space. Therefore, the usable peak performance was above 17 TFLOPs.

**Table 1:** Specifications of all investigated stems

Stem	Manufacturer	Stem-size	Type [1]	Length	max. Depth
Silent®	DePuy®, London, UK	24 × 45	collum	45 mm	24 mm
Metha®	B.Braun Aesculap®, Tuttlingen, Germany	2	partial collum	92 mm	19 mm
Nanos®	Smith&Nephew®, London, UK	2	partial collum	92 mm	17 mm
Aida®	implantcast®, Buxtehude, Germany	0	trochanter sparing	96 mm	16 mm
Fitmore®	Zimmer®, Warsaw, USA	A4	trochanter sparing	93 mm	14 mm
SMF®	Smith&Nephew®, London, UK	1	trochanter harming	90 mm	18 mm
Profemur Preserve®	MicroPort Orthopedics®, Arlington, USA	1	trochanter harming	91 mm	15 mm
Spotorno® *(standard)*	Zimmer®, Warsaw, USA	8	-	146 mm	17 mm

For stress analysis, a weight-dependent head force was applied with a magnitude of 347% of the bodyweight [20, 21]. The vertical axis (z) of the coordinate system was defined by the hip and knee joint centre and the frontal axis (x) by the dorsal aspect of the femoral condyles. So the head force was multiplied by -sin(15°) to obtain the x-component, by -sin(13°) to obtain the y- and by cos(15°) to obtain the z-component [22]. Because of their highly variable in vivo magnitude, simulation of additional muscle forces was disregarded [16, 23].

The strain simulation process after applying the hip center force was performed with the gradient solver (default settings) of the FE software during the solution process. Similar to previously published work, slice by slice analysis was performed before linear analysis with full resolution [24, 25]. In addition, periprosthetic regions of interest (ROI) were defined according to Gruen et al. of each short-stem-type and according to the ROIs of a typical standard stem (CLS Spotorno®, Zimmer, Warsaw, USA) [26].

Statistical analysis was done by a Z-Test according to Paternoster [27] that compares regression parameters. The method estimates adequate fitting curves by different regression models. To find the best fitting curve we test for each type of prostheses a linear, quadratic, and cubic regression function and compare the resulting R^2^ values. The highest R^2^ value is equivalent for the best fit and shows the most adequate model for the underlying relationship. The estimated regression equations that fit the data best for all groups of prostheses are of the form

$$ SED\_{Change}_{ik}={\beta}_{0k}+{x}_{ik}{\beta}_{1k}+{x^2}_{ik}{\beta}_{2k}+{x^3}_{ik}{\beta}_{3k}+{e}_{ik}, $$

with *i* for the observations, *k* for the different groups, and *x* for the bone layers corresponding to the different ROI zones. *e* describes the error term and *β*_0_ − *β*_3_ are the estimated regression parameters that characterise the curves pattern. Correlations between the strain patterns and the length and depth of the implant were analysed by a correlation analysis. The results are calculated with statistical software package SPSS® Version 24 (IBM, Armonk, North Castle, New York, USA).

The Medical Ethics Committee of the University of Marburg approved this study (number of ethical approval: 84/96). Written informed consent was obtained from all study participants before participation.

## Results

**Fig. 2:**
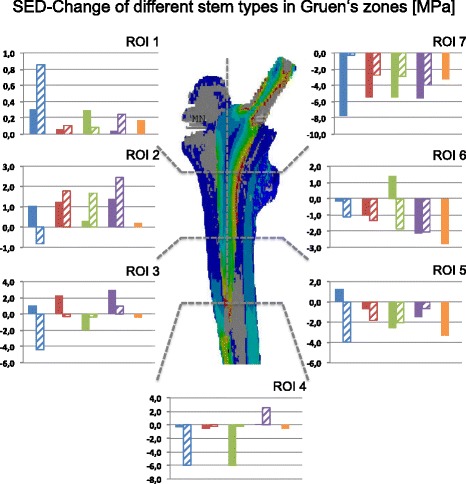
SED-Changes [MPa] in every ROI according to Gruen and colleagues. The different stem types (collum (*blue*), partial collum (*red*), trochanter sparing (*green*), trochanter harming (*violet*), and standard (*orange*)) were taken for stress analysis. ROIs were defined by stem type (populated) and geometry of the standard stem (hatched)

To evaluate the impact of the different short-stem THA-implants on periprosthetic bone structure, a classic strain analysis for every short-stem-type was performed. Figure 2 shows the results of the analysis (strain energy density (SED) change after virtual implantation) for each stem type including a standard reference stem and the native femur without prosthesis. While medial regions of the bone showed clear strain reductions by every stem, the SED in the lateral regions increased in most scenarios. Only in ROI 3 and 4 virtual implantation of the trochanter sparing type stems resulted in a stress-shielding phenomenon leading to lower strain values. In addition, geometrical ROIs of the standard stem (CLS Spotorno®) were taken for analysis in each short-stem scenario to demonstrate the influence of the different stems on a potential revision bone stock. Similar to the findings above, a clear shielding effect appeared only in medial bone regions. The trochanter-sparing effect in ROI 3 and 4 was seen after virtual implantation of the collum type stem. In summary the results demonstrate that implantation of a short-stem prosthesis reduced the stress shielding effect compared to implantation of standard stem.

**Fig. 3:**
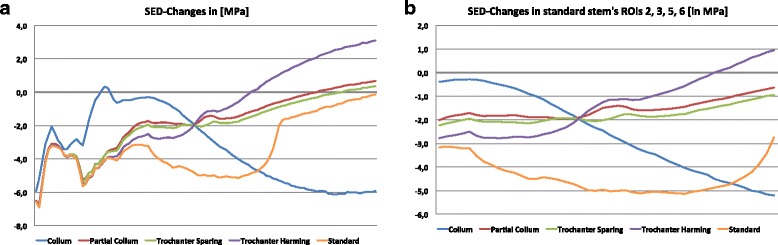
SED-Changes [MPa] from the tip of the trochanter to the end of ROI 4 based on the CLS Spotorno® stem geometry (**a**) and from the tip of ROI 2&6 to the end of ROI 3&5 based on the CLS Spotorno® stem geometry (**b**). The different stem types (collum (*blue*), partial collum (*red*), trochanter sparing (*green*), trochanter harming (*violet*), and standard (*orange*)) were taken for stress analysis

To demonstrate strain patterns in full resolution, a graphical reprocessing of the data set used for the analysis shown in Fig. 3 was performed for the complete bone and for the methadiaphyseal area. Especially in the metadiaphyseal area short stems provide stability for an integration of a standard stem (Fig. 3b). In the trochanteric region, only the collum-type stem showed a biomechanical behaviour more similar to the native femur than any other stem type (Fig. 3a and b). In contrast, partial collum, trochanter harming and trochanter sparing models did not differ between each other for biomechanical behaviour. Furthermore, the three models provided less stress shielding with a lower SED than the standard stem. Additionally, individual data illustration of every stem is shown in Additional file 1 (Figure S3).

To approve our findings shown in Fig. 3, we performed a statistical regression analysis of the stress patterns of every stem-type according to Falez and colleagues. The best fit with regard to the pattern-curves for every stem-type was analysed by a cubic regression line. Fitting curves are available in Additional file 1. To compare the different cubic patterns of the prostheses, a Z-test for pairwise comparisons was performed. Table 2 shows the calculated z-values for the differences of the regression parameters. The results show that the majority of the stem-types have different SED change patterns. Only the collum and standard type as well as the partial collum and the trochanter sparing type do not differ from each other. Thus, for this stem-types the progress of SED change is equal and can be estimated with the same regression parameters keeping in mind that they start from different initial levels.

**Table 2:** Statistical testing

	***β***_1***k***_ − ***β***_1***k***_	***β***_2***k***_ − ***β***_2***k***_	***β***_3***k***_ − ***β***_3***k***_
*Collum* vs. *trochanter* s*paring*	5.958***	−7.109***	5.961***
*Collum* vs. *trochanter harming*	9.651***	−10.456***	9.263***
*Collum* vs. *standard*	1.864	−0.656	−0.246
*Partial collum* vs. *trochanter sparing*	−0.217	0.247	−0.245
*Partial collum* vs. *trochanter harming*	4.997***	−5.063***	4.6434***
Partial collum vs. standard	−1.8472	3.517***	−4.575***
*Trochanter sparing* vs. *trochanter harming*	5.600***	−5.696***	5.238***
*Trochanter sparing* vs. *standard*	−1.840	3.643***	−4.803***
*Standard* vs. *trochanter harming*	6.109***	−7.613***	8.149***

*** z ≥ 3.29 (two sided)

Additionally, the correlation analysis shows no significant impact of the stem-length (*p* = 0.961) and the stem-depth (*p* = 0.243) (Table 1) on the overall change of SED. In summary the analysis demonstrated that the SED did not differ after implantation of partial collum, trochanter sparing, or trochanter harming stems.

## Discussion

Currently, there are multiple designs of short-stem THA-prostheses available [2]. Different fixation-techniques defined by osteotomy level are supposed to provide a maximum of bone stock preservation until the first aseptic revision surgery [9, 28]. A surgeon’s decision for a special stem design is often complicated by missing biomechanical data. The main objective of this study was to compare stems-designs of THA-prostheses for their impact on stress-shielding of the periprosthetic bone. Our findings show a stress-shielding prevention by common short-stems independent from the level of osteotomy.

In the current study, we used the previously validated FEA based on clinical data to show the impact of a short-stem THA-implant on stress shielding and bone remodelling [6, 7, 15]. Descriptive examinations can be performed by dual X-ray absorptiometry (DEXA), a technique that operates with lower radiation-doses than CT-based methods. However, DEXA could not be used for simulations in this study because it lacks several virtual implantation options. The transfer from bone density values to elastic modulus has been performed by different mathematical approaches [17, 29–32]. According to studies from Ciarelli and colleagues, it was possible to use a linear relationship because FE models provide consistent bony stress-shielding patterns and are independent of the used density-modulus-relationship [16, 17]. Because an identical specimen dataset was used for each simulation, common problems of a CT-based approach such as partial volume effects, fat errors, or metal artefacts had no impact on our conclusions. Because in the used model, the influence of muscle forces on the SED was not exactly clear, the applied hip forces were verified by telemetric in vivo measurements [33]. This led us to choose a simulation of an isolated resultant force on the centre of the hip joint.

Over decades most biomechanical THA studies used a classification of seven delineated sections (ROI) for quantification of zonal radiographic bone looseness described by Gruen and colleagues in 1979 [26]. Their study was based on a first essay of Salvati and colleagues from 1976 [34]. Gruen’s classification is used for manual radiographic analyses and ensures a worldwide comparability of radiologic conclusions until today. Since in the digital era new powerful tools like computerized DEXA or CT-based data sets have arisen, it becomes easy to provide high-resolution results. Therefore, Joshi and colleagues used an approach with twelve ROIs [11]. In addition to this, we performed an analysis slice by slice taking into account the complete information without data compression. With this approach, we can provide full slice resolution with linear analysis.

In concordance with previous studies, results did not show a significant change of the periprostetic bone strain based on the length of the stem. In addition, a greater proximal dimension of the stem in the sagittal plane was not associated with any reduction in periprostetic bone strain [9, 35].

Falez and colleagues have provided a viable classification of different short-stem designs of THA-implants with regard to the level of osteotomy [9]. The intention of a force application as proximal as possible trough the femoral stem led to many developments on the prostheses market. Starting with a “hip resurfacing arthroplasty” (e.g. Burmingham Cap® model), multiple efforts were made to develop new hip stems with a reduced stress-shielding effect [14, 36, 37]. The “collum type” represents a stem type that can be implanted as proximal as possible behind a cap model. However, common complications like osteonecrosis or pitfalls during the surgical procedure led to an early fail of those implants. Therefore, most of the above mentioned stems are no longer available [35, 38]. Like others, our findings for the proximal section describe a strain energy loading close to a native bone. However, the middle and the distal section do not profit by this stem type [35]. Neither in our results nor in findings from other study groups, the advantage of a collum-type prosthesis in the proximal section could be reproduced by metaphyseal anchoring stems [6, 12].

Therefore, representative stems of the partial collum, the trochanter harming and the trochanter sparing types were studied clinically and biomechanically [8, 39–41]. Like in previous studies, we could demonstrate a reduced stress shielding effect of stort-stem implants in comparison to a standard stem implants [8, 39–42]. Similar to Götze and colleagues who detected a non-physiological strain loading by a partial collum type stem, our findings suggest that there is no advantage of force application located most proximally compared to an osteotomy approach located most distally [8]. Floerkemeier and colleagues could show as well that a lower resection even due to more similar strain patterns to a femur model without an implant which suggest that a maximum bone preservation at the femoral neck do not lead to a more physiological load [43]. However, in case of revision surgery, the metadiaphysal bone stock is relevant for optimal ingrowth of a secondary THA-implant using a standard stem design. For that case, an approach with a distal osteotomy is associated with a reduced stress-shielding effect. Similarly, Heller and colleagues could show that changes in stem positioning of short stem implants had no influence on stress shielding effects or the short-stem itself [44].

Using cubic patterns for mathematical description of the strain load into the femur bone, the results showed similar regression slopes for collum-type and standard prostheses indicating similar bone loss pattern for both stem types. However, it is important to note that the regression slopes only capture patterns of SED changes and not absolute differences. In addition, a difference between the partial collum and trochanter sparing type was not observed.

The current study is limited by the virtual approach of THA stem implantation. It is unclear how data obtained from implantation simulations with FE software translate into the clinical setting. Our findings should be validated in DEXA or cadaver studies as a next step to obtain further data on the biomechanical physiology of the different stem types. This information might be a prerequisite for design of a prospective clinical trail.

## Conclusion

Based on the result of this study, we cannot confirm that the design of current short stem THA-implants leads to a different stress shielding effect with regard to the level of osteotomy. Somehow unexpected, we found a bone stock protection in metadiaphyseal bone by simulating a more distal approach for osteotomy. In the proximal bone only a collum THA-prosthesis could lead to this effect but so far there is no THA-implant on the marked that provides this kind of stem. Further clinical and biomechanical research including long-term results is needed to understand the influence of short-stem THA on bone remodelling and to find the optimal stem-design for reduction of the stress-shielding effect.

## Additional files

Additional file 1: Supplementary data are available in the file “Supplement_BMSD-D-17-00428 (12891_2017_1702_MOESM1_ESM)” in the online Data Supplement. Supplementary data provide: 1) details on statistical analysis, 2) a comparison of SED Changes (from the tip of the trochanter to the end of ROI 4 based on the CLS Spotorno® stem geometry) of all 8 analysed stems. (DOCX 9154 kb)
