# Supplementary material for: Bone preserving level of osteotomy in short-stem total hip arthroplasty does not influence stress shielding dimensions – a comparing finite elements analysis
Source: BMC Musculoskelet Disord. 2017 Aug 7;18:343. doi: 10.1186/s12891-017-1702-2 (PMC5545828; doi:10.1186/s12891-017-1702-2)
Supplement: Supplementary file 1 — Supplementary data are available in the file “Supplement_BMSD-D-17-00428 (12891_2017_1702_MOESM1_ESM)” in the online Data Supplement. Supplementary data provide: 1) details on statistical analysis, 2) a comparison of SED Changes (from the tip of the trochanter to the end of ROI 4 based on the CLS Spotorno® stem geometry) of all 8 analysed stems. (DOCX 9154 kb) [file 12891_2017_1702_MOESM1_ESM.docx]

**Additional file 1**

**Bone preserving level of osteotomy in short-stem total hip arthroplasty does not influence stress shielding dimensions – a comparing finite elements analysis**

Rene Burchard, Sabrina Braas, Christian Soost, Jan Adriaan Graw, Jan Schmitt

**Statistics**

Adequate fitting curves were estimated with different regression models. For each prosthesis group a linear, a quadratic, and a cubic regression function were tested and resulting R^2 values were compared. The highest R^2 value equals the best fit and provided the best model for the underlying relationship.

The estimated regression equation with the best data fitting for all groups of prostheses appears as follows:

${SED\_Change}_{ik}=\beta_{0k}+x_{ik}\beta_{1k}+{{x^{2}}_{ik}\beta}_{2k}+{{x^{3}}_{ik}\beta}_{3k}+e_{ik}$,

$i$ stands for observations, $k$ for the different groups, and $x$ for the bone layers corresponding to the different ROI zones. The variable $e$ stands for the error term and $\beta_{0}-\beta_{3}$ are the estimated regression parameters that characterize the curve patterns

For each prosthesis group we observed the cubic regression line as the best fit. The fitting curves are presented below (Supplemental Figure 1-5). SED-Changes [MPa] from the tip of ROI 2&6 to the end of ROI 3&5 are based on the CLS Spotorno® stem geometry.


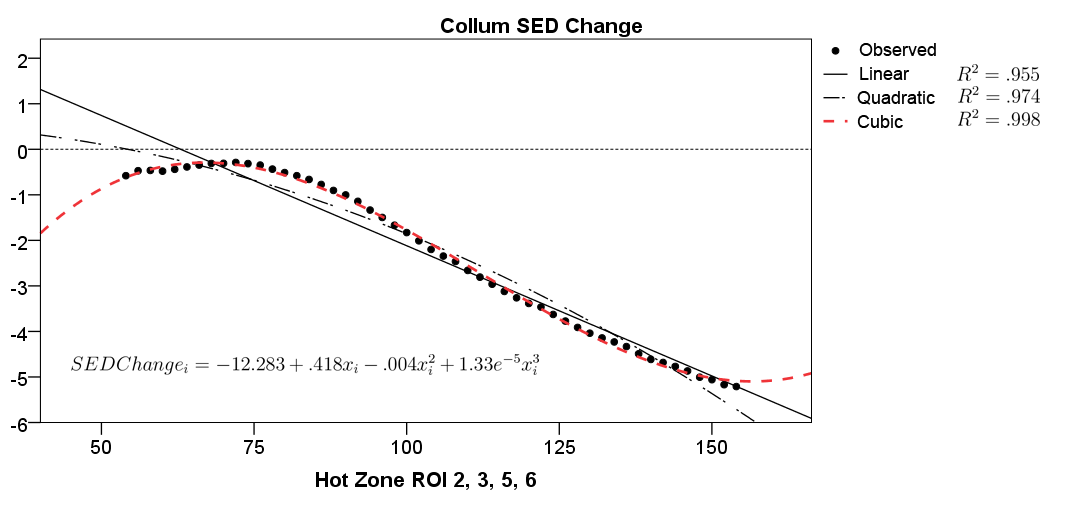


**Figure S1.** Fitting curve of the collum type stems in the relevant ROIs referring a standard stems’ bone stock.


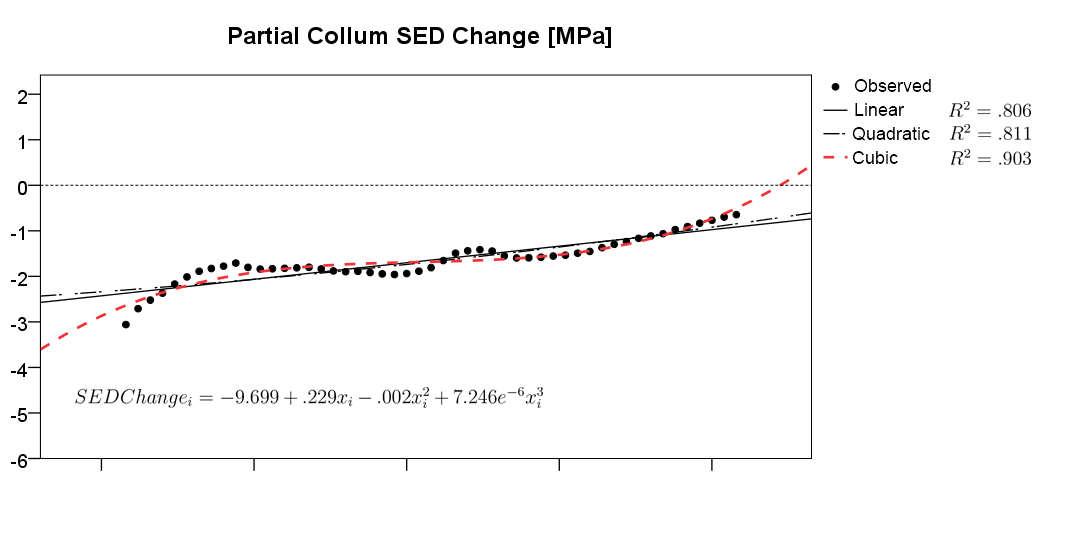


**Figure S2.** Fitting curve of the partial collum type stems in the relevant ROIs referring a standard stems’ bone stock.


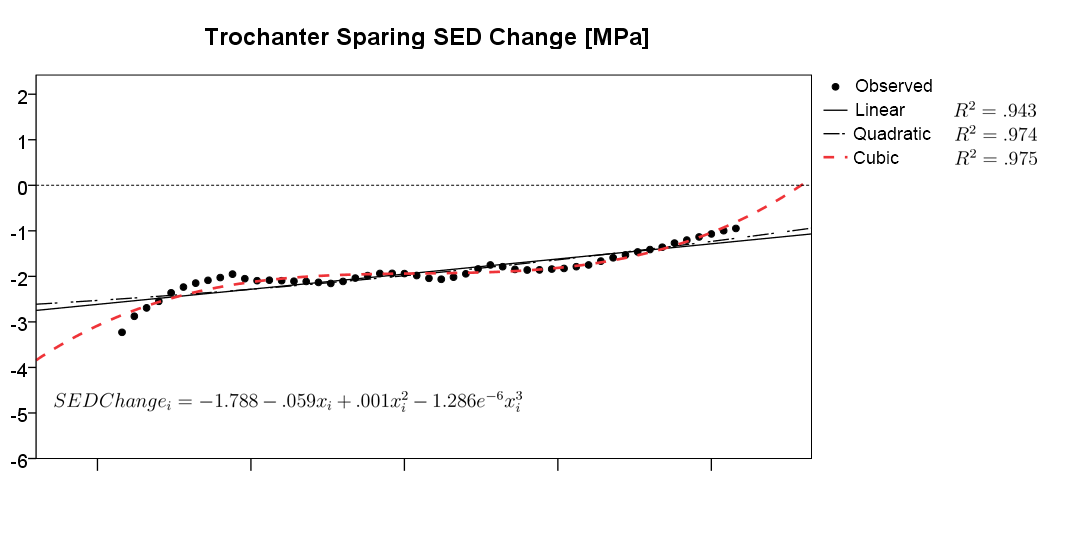


**Figure S3.** Fitting curve of the trochanter sparing type stems in the relevant ROIs referring a standard stems’ bone stock.


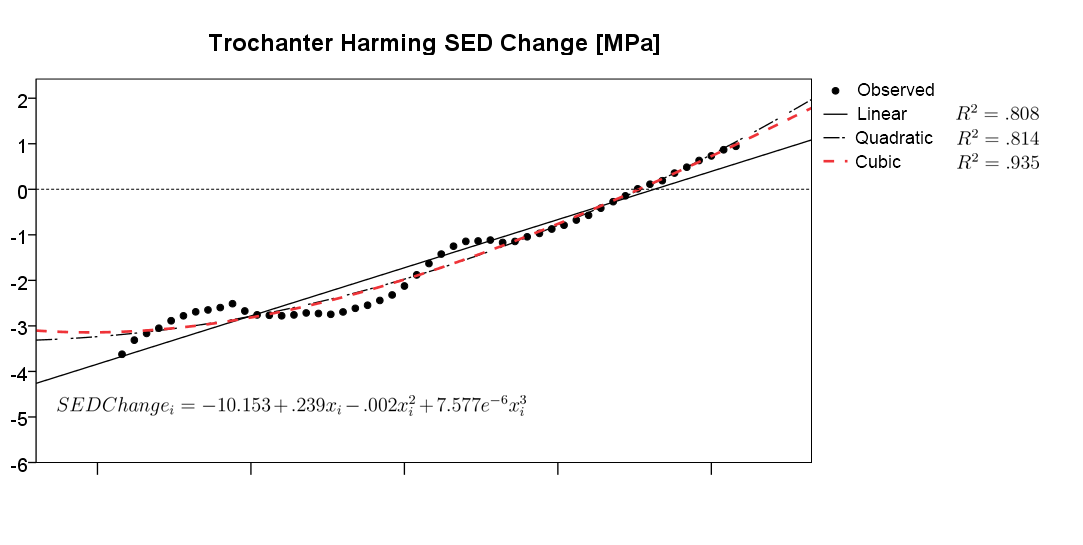


**Figure S4.** Fitting curve of the trochanter harming type stems in the relevant ROIs referring a standard stems’ bone stock.


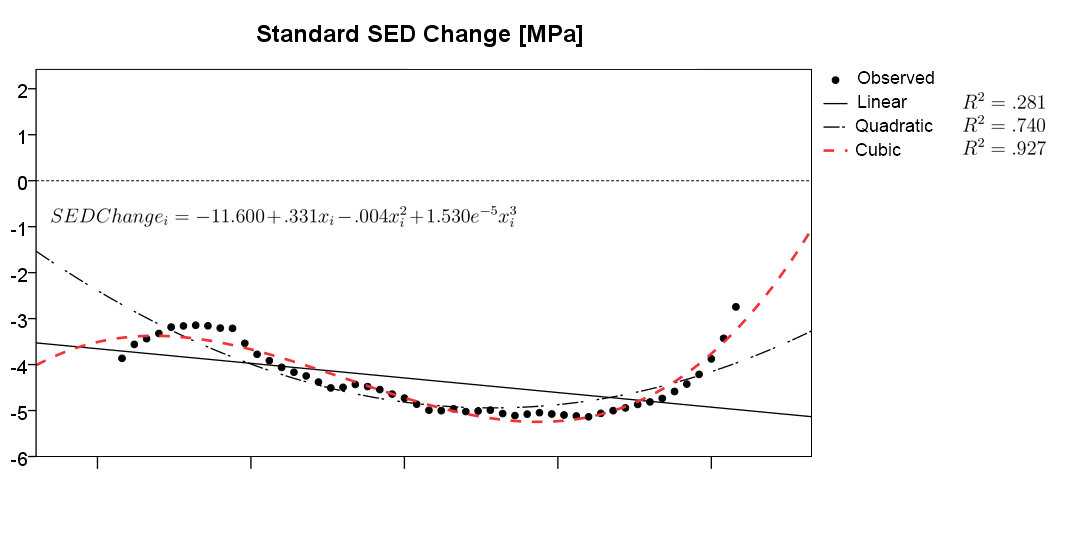


**Figure S5.** Fitting curve of the standard type stems in the relevant ROIs referring a standard stems’ bone stock.

**Figure S6. -** **SED-Changes [MPa] from the tip of the trochanter to the end of ROI 4 based on the CLS Spotorno® stem geometry.** The different stems (Silent® (middle blue), Nanos® (orange), Metha® (grey), Fitmore® (yellow), AIDA® (dark blue), SMF® (green), Profemur® (light blue), and CLS Spotorno® (light orange)) were taken for stress analysis.
